# m6A-related metabolism molecular classification with distinct prognosis and immunotherapy response in soft tissue sarcoma

**DOI:** 10.3389/fimmu.2022.895465

**Published:** 2022-07-28

**Authors:** Zhen-Dong Huang, Yong-Cheng Fu, Shu-Yan Liu, Ya-Juan Mao, Yan Zhang, Chao Hu, Ren-Xiong Wei

**Affiliations:** ^1^ Department of Spine and Orthopedic Oncology, Zhongnan Hospital of Wuhan University, Wuhan, China; ^2^ School of Stomatology, Southern Medical University, Guangzhou, China; ^3^ The Third Clinical School, Hubei University of Medicine, Shiyan, China

**Keywords:** m6A modification, cancer metabolism, tumor microenvironment, molecular classification, machine learning, immunotherapy

## Abstract

N6-methyladenosine (m6A) methylation, one of the most crucial RNA modifications, has been proven to play a key role that affect prognosis of soft tissue sarcoma (STS). However, m6A methylation potential role in STS metabolic processes remains unknown. We comprehensively estimated the m6A metabolic molecular subtypes and corresponding survival, immunity, genomic and stemness characteristics based on 568 STS samples and m6A related metabolic pathways. Then, to quantify the m6A metabolic subtypes, machine learning algorithms were used to develop the m6A-metabolic Scores of individual patients. Finally, two distinct m6A metabolic subtypes (Cluster A and Cluster B) among the STS patients were identified. Compared to Cluster B subtype, the Cluster A subtype was mainly characterized by better survival advantages, activated anti-tumor immune microenvironment, lower gene mutation frequency and higher anti-PD-1 immunotherapy response rates. We also found that the m6A-metabolic Scores could accurately predict the molecular subtype of STS, prognosis, the abundance of immune cell infiltration, tumor metastasis status, sensitivity to chemotherapeutics and immunotherapy response. In general, this study revealed that m6A-regulated tumor metabolism processes played a key role in terms of prognosis of STS, tumor progression, and immune microenvironment. The identification of metabolic molecular subtypes and the construction of m6A-metabolic Score will help to more effectively guide immunotherapy, metabolic therapy and chemotherapy in STS.

## Introduction

Soft tissue sarcoma (STS) constitutes more than 50 subtypes of malignant tumors that originate from the interstitial connective tissues (1). The main treatment options for patients with STS include surgical resection, radiotherapy, and chemotherapy. However, metastasis or recurrence is reported in approximately 50% of STS patients after treatment (2). Furthermore, the median survival time of patients with metastatic STS is only 8-12 months (3). Immunotherapy is a promising treatment for several solid tumors. The survival benefits of immune checkpoint inhibitors are higher in several cancers compared to chemotherapy and targeted therapy (4). Moreover, immunotherapies such as IL-15 cytokine therapy in combination with TIGIT blockade therapy (5) as well as PD-1 and CTLA-4 inhibitors (6) have made progress in treating patients with STS. However, the complexity and heterogeneity of tumor microenvironment (TME) affects the efficacy of immunotherapies in STS patients. Currently, the immunotherapy response rate in patients with STS is significantly low (7). Therefore, more precise molecular classification is urgently needed to unravel the TME heterogeneity of STS to select patients more suitable for immunotherapy and improve the response rate of immunotherapy.

N6-methyladenosine (m6A) is the most abundant and common epigenetic modification in the eukaryotic mRNAs. The protein machinery involved in m6A recognition, addition, or removal, including m6A methyltransferases (writers), m6A demethylases (erasers), and m6A readers, has been well characterized. Several reports have shown that alterations in m6A mRNA methylation disrupt gene expression and the downstream cellular processes, and play a significant role in the initiation and progression of tumors (8). Dysregulation of m6A mRNA methylation in the cancer cells alters the expression of metabolic genes and the activities of the related metabolic pathways, thereby significantly affecting proliferation, differentiation, invasion, and metastasis of cancer cells (9, 10). Metabolic pathways play a crucial role in tumorigenesis, cancer cell survival, and regulation of the tumor immune microenvironment (TIME) (11). Therefore, characterization of the m6A-related metabolic signatures in the STS tissues can unravel the status of the tumor microenvironment (TME) and help the clinicians to strategize immunotherapy options for individual patients. Recent study has shown that molecular typing of STS tumors is more accurate than the traditional classification of STS tumors based on pathological staging and TNM staging (10). Therefore, in this study, we integrated 568 STS samples from the TCGA-SARC and GSE21050 cohorts, and comprehensively evaluated the association between m6A-related metabolic pathways and immune characteristics of STS using multi-omics data. We also analyzed the different molecular subtypes of STS based on m6A-related metabolism. Furthermore, we established a scoring system based on m6A-related metabolism and evaluated its accuracy in predicting the prognosis and immunotherapy response of STS patients.

## Materials and methods

### STS dataset acquisition and processing

RNA-sequencing data were downloaded for the STS samples in the TCGA-SARC cohort (https://gdc.xenahubs.net) in the form of normalized Fragments Per Kilobase of transcript per Million mapped reads (FPKM). Then, the FPKM values were then transformed into transcripts per kilobase million (TPM) values. The microarray data for the STS samples from the GSE21050 cohort were downloaded from the Gene Expression Omnibus (GEO) database (https://www.ncbi.nlm.nih.gov/geo/). The TCGA and GEO datasets (n=568) were merged and the batch effect biases were corrected using the “ComBat” algorithm based on R package “sva”. All statistical analyses were conducted using R software 4.1.2 (https://www.r-project.org/).

### Pre-processing clinicopathological and genomic data

Clinicopathological data, including overall survival rates of the STS patients in the TCGA-SARC and GSE21050 cohorts, were extracted. The baseline clinicopathological characteristics of the STS samples and immune microenvironment tissue classification (12) are shown in **Supplementary Table S1**. Genomic data, including somatic mutations and copy number alterations (CNA) of the TCGA-SARC cohort, were obtained from the Firehose project (https://gdac.broadinstitute.org/). The mutational landscape of the STS samples was visualized using the “maftools” R package (13). The arm-level and focal-level genome amplifications and deletions were analyzed using the GISTIC 2.0 package based on GenePattern tool (https://cloud.genepattern.org) (14).

### Metabolic pathway acquisition and unsupervised clustering

We acquired 114 gene sets related to tumor metabolism from previously published study (15). The single sample Gene Set Enrichment Analysis (ssGSEA) algorithm was used to estimate the enrichment scores of various metabolic pathways based on gene expression profiles. Each sample was assigned a score corresponding to the status of each metabolic pathways. Next, we identified 21 m6A regulators from previous studies (16), including 11 m6A readers (*ELAVL1, FMR1, HNRNPA2B1, HNRNPC, IGF2BP1, LRPPRC, YTHDC1, YTHDC2, YTHDF1, YTHDF2*, and *YTHDF3*), 8 m6A writers (*CBLL1, KIAA1429, METTL14, METTL3, RBM15, RBM15B, WTAP*, and *ZC3H13*), and 2 m6A erasers (*ALKBH5* and *FTO)*. Pearson’s correlation analysis was used to identify m6A-related metabolic pathways using |Pearson’s r| >0.4 and p <0.001 as the criteria. Univariate Cox regression analysis was used to identify the m6A-related metabolic pathways closely related to the prognosis of STS patients. The m6A-related metabolic pathways were ranked according to their degree of importance to STS patient prognosis (nrep = 100 iterations in the Monte Carlo simulation; nstep = 5) using the random survival forest (RSF) algorithm. Then, a network to demonstrate the interactions between the m6A-related metabolic pathways was constructed based on Spearman and distance correlation analyses.

The unsupervised clustering analysis was performed using the Consensus Clustering algorithm to identify clusters based on distinct statuses of the m6A-related metabolic pathways associated with prognosis. The STS samples were classified by k-means, with k from 2 to 10 using the ”ConsensusClusterPlus” R package (17) and 1000 repetitions were performed to ensure clustering stability. The consensus clustering matrix and cumulative distribution function (CDF) curve analysis was used to determine the optimal number of clusters.

### Tumor immune microenvironment analysis

The abundance of immune cell types in the TIME was determined using the “immunedeconv” and “gsva” R packages (18, 19), which estimated immune cell infiltration based on seven different algorithms, namely, ssGSEA, TIMER, xCell, MCP-counter, EPIC, quanTIseq, and IPS. “ESTIMATE” R package (20) was then used to determine the immune scores and tumor purity of different molecular subtypes of STS patients.

### Gene set variation analysis and functional enrichment analysis

The pathway enrichment profiles of different clusters were evaluated using the “gsva” R package. The enrichment scores of twelve well-defined tumor-related gene sets or pathways (**Supplementary Table S2**) were calculated for all samples using the GSVA algorithm. Gene ontology (GO) enrichment analysis was performed with the “clusterProfiler” R package (21) using q-value <0.05 as the cutoff.

### Calculation of stemness index

The one-class logistic regression (OCLR) machine learning algorithm (22) was used to calculate the mRNA expression-based stemness index (mRNAsi) of the tumor samples. The mRNAsi value indicated the stem cell-like features of the cancer cells in the samples. We then compared the differences in stemness levels (mRNAsi) between the clusters using Wilcoxon test. P values of less than 0.05 were considered statistically significant.

### Evaluation of the m6A-metabolic score

Next, we evaluated the m6A-metabolic Score for STS patients. First, we identified the differentially expressed genes (DEGs) using log2 fold change (FC) >1.5 and adjusted P-value <0.05 as the criteria between the between clusters based on the status of the m6A-related metabolic pathways using “limma” R package (23). Then, the unsupervised clustering algorithm consensus clustering was used to assign STS patients into different gene clusters based on the DEGs. The m6A-related metabolism scoring system was developed using the least absolute shrinkage and selection operator (LASSO) algorithm based on the “glmnet” R package with 10-fold cross validation to estimate the penalty parameters (24). The m6A-metabolic Score was calculated using the following formula: m6A-metabolic $\text{Score}=\sum_{i=1}^{n}\text{Coefficient}*\text{Exp}$ , where Exp is the expression value of each selected hub gene from LASSO algorithm. Then, we identified hub genes as independent prognostic factors using multivariate Cox regression analysis. We then developed a clinical nomogram to predict the survival of STS patients by integrating the clinicopathological features and m6A-metabolic scores using the “rms” R package.

The cutoff value was determined based on the correlation between the m6A-metabolic score and prognosis using the “surv-cutpoint” function of the “survminer” R package. The STS patients were classified into groups with high- and low-m6A-metabolic Scores based on the cut-off value.

### Immunotherapy response prediction

The expression levels of 14 immune checkpoint-related genes were used to predict the immunotherapy response of each STS patient. Next, the Tumor Immune Dysfunction and Exclusion (TIDE) algorithm was used to predict responses to immune checkpoint blockade therapy (25). The tumor immune evasion characteristics of the pre-treatment tumor profiles were derived by integrating the expression signatures of T cell dysfunction and T cell exclusion in the tumor tissues. Furthermore, the “tumor inflammation signature” (TIS) score consisted of 18 key gene signatures that were estimated to quantify immune response activation in the TIME (26). TIS score was then used to predict response to anti-PD-1 immunotherapy. In general, a lower TIDE score or a higher TIS score correlated with better response to immunotherapy. The correlation between m6A-related metabolism score, TIDE score, and TIS score was analyzed and the differences in the TIDE scores and TIS scores between the high and low m6A-metabolic score groups was compared.

The Subclass Mapping (SubMap) algorithm (27) was used to compare the similarities in the expression profiles of the high- and low-m6A-metabolic Score groups among the STS cohorts and a clinical cohort with 47 melanoma patients treated with immunotherapy (28). The lower the p values, the higher the similarity. Recommended default parameters, 1000 random permutations for Fisher’s statistics, were used. The “complexHeatmap” R package (29) was used to visualize the SubMap results.

### Chemotherapy response prediction

The chemotherapy response was predicted for each STS sample using the predictive model for the cell line data from the publicly available Genomics of Drug Sensitivity in Cancer (GDSC) database (https://www.cancerrxgene.org/). A lower half-maximal inhibitory concentration (IC50), estimated by ridge regression, indicates a better sensitivity to a given drug. IC50 of a drug was estimated by ridge regression to determine the degree of sensitivity. The chemotherapeutic response prediction was performed using the “pRRophetic” R package (30).

## Results

### Eleven prognostic m6A-related metabolic pathways are identified in the STS cohort

**Figure 1:**
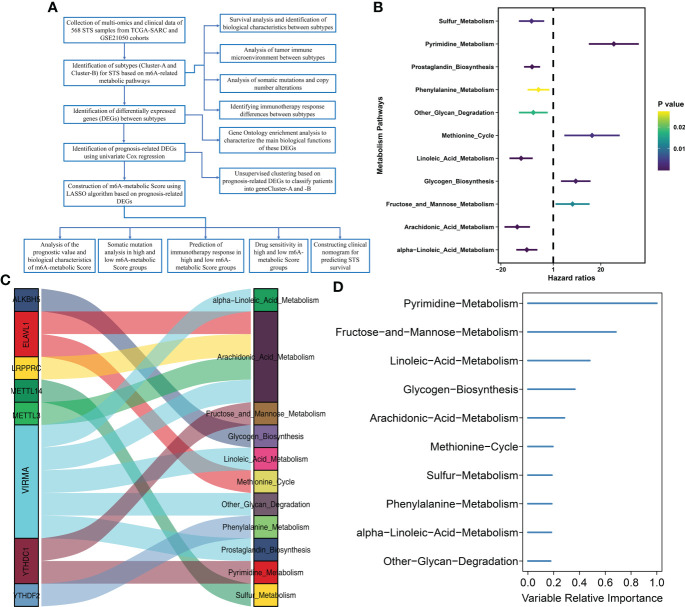
Flow diagram and prognostic m6A-related metabolic pathways are identified **(A)** Flow diagram shows this study’s systematic analysis process. **(B)** Univariate Cox regression analysis reveals 11 m6A related metabolic pathways significantly correlated with STS prognosis **(C)** The alluvial diagram displays 8 m6A regulators and 11 m6A-related metabolic pathways. **(D)** 11 m6A related metabolic pathways were ranked by Random Survival Forests algorithm.

The study design and strategy are shown in **Figure 1A**. To reduce the likelihood of batch effects from non-biological technical biases, the “ComBat” algorithm was used between TCGA-SARC and GSE21050 datasets (**Supplementary Figure S1**). The ssGSEA algorithm was used to analyze the status of 114 metabolic pathways in the STS samples. The metabolic pathways were assigned scores for every STS sample enrolled in this study. Then, 35 m6A-related metabolic pathways were identified by evaluating 21 m6A regulators (8 writers, 2 erasers, and 11 readers) and the 114 metabolic pathways using Pearson correlation analysis (**Supplementary Table S3**). Univariate Cox regression analysis showed that 11 m6A-related metabolic pathways were significantly associated with the prognosis of STS patients. These 11 metabolic pathways were used for subsequent analysis. **Figure 1B** shows the prognostic values of the 11 m6A-related metabolic pathways in the STS patients. A total of 8 m6A regulators (*VIRMA, METTL3, LRPPRC, ELAVL1, YTHDC1, ALKBH5, YTHDF2, METTL14*) associated with 11 metabolic pathways that play a key role in the prognosis of STS were screened, and their co-expression networks were showed in **Figure 1C**. These 11 m6A-related metabolic pathways were ranked using the RSF algorithm. Pyrimidine metabolism, fructose and mannose metabolism, and linoleic acid metabolism were the top 3 m6A-related metabolic pathways (**Figure 1D**).

### Identification and validation of two distinct molecular subtypes of STS based on m6A-related metabolic pathways

**Figure 2:**
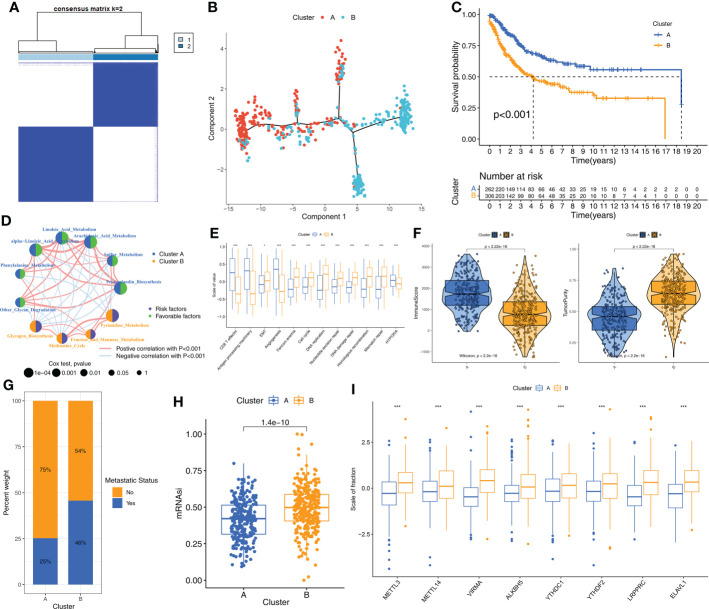
The molecular subtypes based on prognostic m6A-related metabolic pathways **(A)** The consensus matrix heatmap corresponding to k=2 obtained by consensus clustering. **(B)** STS patients could be stratified into two subtypes based on m6A-related metabolic pathways; each point represents a patient with colors corresponding to two subtypes. **(C)** Kaplan–Meier curves for overall survival of 568 STS patients from TCGA-SARC and GSE21050 cohorts. Log-rank test revealed P-value <0.001. **(D)** The interactions of 11 m6A-related metabolic pathways in STS. The size of the circles indicated the effect of each m6A-related metabolic pathway on prognosis, respectively. Prognostic risk factors were shown in purple and prognostic favorable factors were shown in green. The lines connecting m6A-related metabolic pathways represented the interactions between m6A-related metabolic pathways, and the thickness of the lines represented the correlation strength estimated by Spearman correlation analysis. Red was a positive correlation, and blue was a negative correlation. **(E)** Differences in immune-related and cancer-related signatures between Cluster A and B. The asterisks represented the statistical p value (*P < 0.05; ***P < 0.001). **(F)** Violin plot shows the difference of ImmuneScore and tumor purity from ESTIMATE algorithms in Cluster A and B. **(G)** The proportion of STS patients with metastatic status in TCGA-SARC and GSE21050 cohorts. **(H)** The box plot shows differences in stemness index (mRNAsi) between Cluster A and B. The statistical difference of clusters was compared through the Wilcoxon test. **(I)** The difference of expression for eight m6A regulators associated with metabolic pathways between Cluster A and B. Wilcoxon test was used to test statistical difference (***P < 0.001).

Consensus clustering was performed to identify the molecular subtypes of STS patients based on the prognostic m6A-related metabolic pathways. K=2 was selected as the optimal number of clusters based on consensus matrix and the cumulative distribution function (CDF) plots (**Figures 2A**, **Supplementary Figure S2**). STS patients were classified into two subtypes, Cluster A (n=262) and Cluster B (n=306). The graph learning-based dimensionality reduction technique was used to distribute individual STS patients into specific branches based on the expression profiles of the prognostic m6A-related metabolic pathways. This analysis also classified STS patients into two groups that were consistent with the defined molecular subtypes (**Figure 2B**). Furthermore, clustering analysis was performed separately on the TCGA-SARC and GSE21050 cohorts as the validation datasets. The results again confirmed the stability of the K=2 clusters (**Supplementary Figure S3A**). Kaplan–Meier survival analysis demonstrated that the overall survival (OS) rates of STS patients in Cluster A was significantly higher than those in Cluster B (**Figure 2C**). The heatmap in **Supplementary Figure S3B** shows the differences in the scores of the m6A-related metabolic pathways in the STS patients belonging to clusters A and B. The interaction network between the 11 prognostic m6A-related metabolic pathways and their prognostic value were shown in **Figure 2D**. These results demonstrated significant differences in the survival rates and the metabolic characteristics of the STS patients belonging to these two molecular subtypes.

### STS samples in Cluster A subtype demonstrate higher anti-tumor immunity than those in Cluster B subtype

Next, we investigated the differences in the biological characteristics of the two subtypes to determine the mechanisms underlying the differences in prognosis. The evaluation of 11 cancer-related pathways (CD8^+^ T effector, Antigen processing machinery, Epithelial-Mesenchymal Transition [EMT], Angiogenesis, Cell cycle, DNA replication, Nucleotide excision repair, DNA damage repair, Homologous recombination, Mismatch repair, Hypoxia) using the GSVA algorithm showed that pathways related to immune activation were significantly up-regulated in the STS samples from Cluster A, whereas, pathways related to EMT, cell cycle, and DNA replication were significantly up-regulated in the STS samples from Cluster B (**Figure 2E**). Next, the differences in the TIME and the status of immune cell infiltration were analyzed in the STS tissues from the two clusters using seven algorithms, namely, ssGSEA, MCPcounter, xCell, EPIC, TIMER, quanTIseq, and IPS. STS samples in Cluster A showed significantly higher infiltration of antitumor immune cell types such as dendritic cells, CD8^+^ T cells, and cytotoxic T cells compared to the STS samples in Cluster B (**Supplementary Figure S3C**). The immune scores were higher for the STS samples in Cluster A compared to the samples in Cluster B, whereas, tumor purity was higher for the STS samples in Cluster B compared to the STS samples in Cluster A (**Figure 2F**). Cluster B samples also showed higher degree of tumor metastases (**Figure 2G**) and stemness levels (mRNAsi) compared to the Cluster A samples (**Figure 2H**). Furthermore, the m6A regulators were up-regulated in the Cluster B samples compared to the Cluster A samples (**Figure 2I**). Overall, these results showed that STS samples in Cluster A exhibited higher anti-tumor TIME than the STS samples in Cluster B.

### Cluster B subtype shows higher genomic mutations and alterations than Cluster A subtype

**Figure 3:**
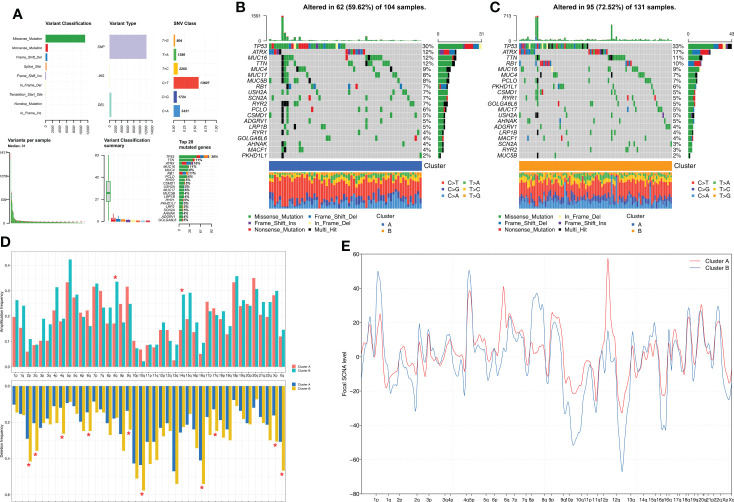
Comparison of somatic mutation and CNA analysis between Clusters. **(A)** The summary of the overall distribution of mutation in STS. **(B, C)** The oncoPrint plots show tumor somatic mutation landscape between Cluster A subtype **(B)** and Cluster B subtype **(C)**. **(D, E)** Comparisons of arm-level amplification and deletion frequencies and focal-level amplification and deletion levels between Cluster A and Cluster B *P < 0.05.

Next, we investigated the differences in somatic mutations and CNA between the two molecular subtypes of STS. Among the somatic mutations, missense mutations and single nucleotide polymorphisms (C>T) were the most common mutation types in the STS samples. The five most frequently mutated genes were *TP53, TTN, ATRX, MUC16*, and* MUC4* (**Figure 3A**). The mutation landscape for the two clusters was displayed using the oncoPrint plot. The mutation frequency was significantly higher for the Cluster B samples (72.52%) compared to the Cluster A samples (59.62%) (**Figures 3B**, **C**). Among the commonly mutated genes, the mutation frequency of *TP53* was highest in both groups. STS patients from both clusters exhibited significant differences in the frequency of deletions at the arm level. Overall, the amplification and deletion frequencies were significantly higher in the Cluster B samples at both the arm-level and the focal-level compared to the Cluster A samples (**Figure 3D, E**).

### Cluster A patients show better response to immunotherapy than the Cluster B patients

**Figure 4:**
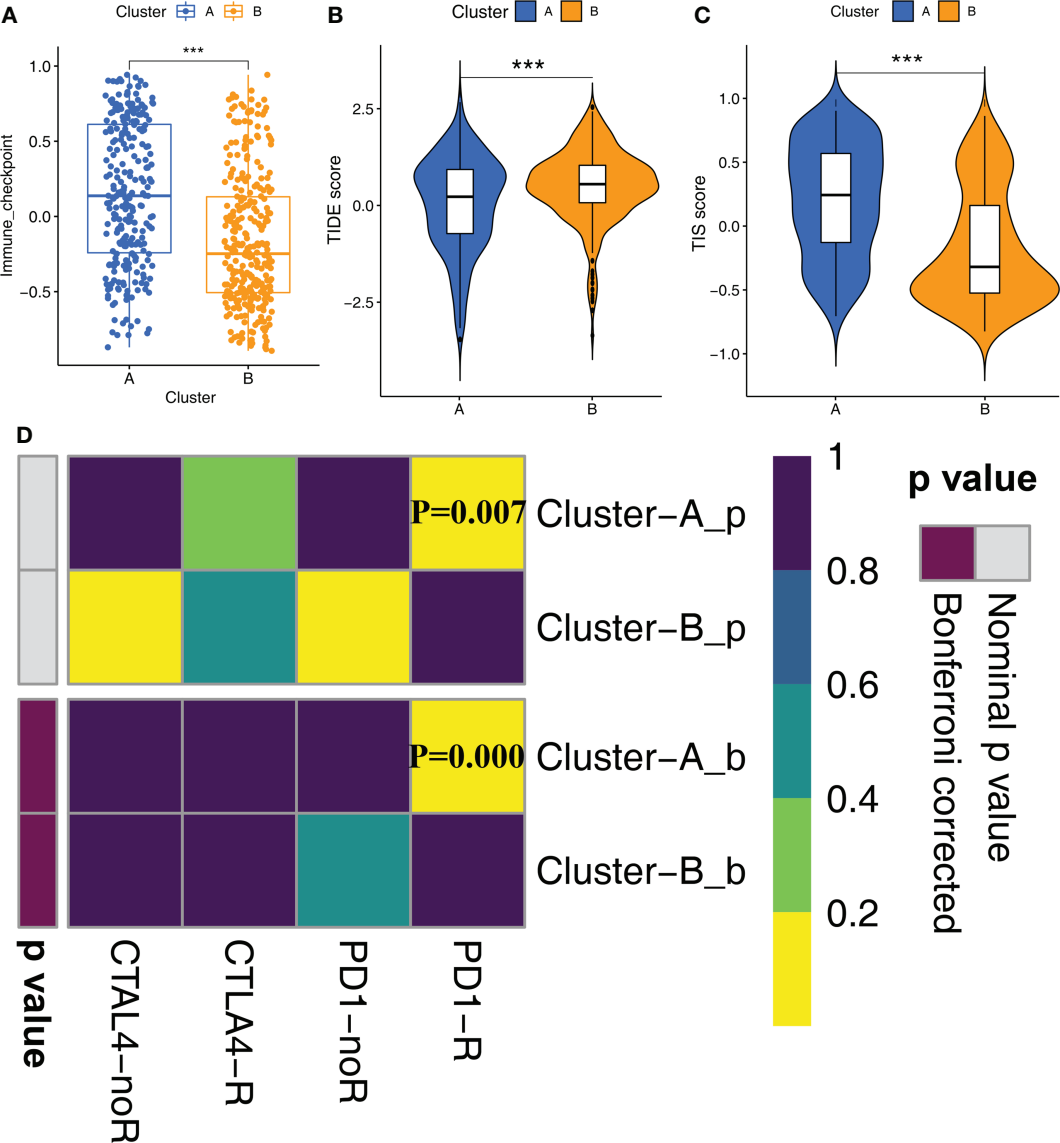
Immune checkpoint inhibitor therapy responses between Clusters **(A)** The differences in the scores of immune checkpoint gene set between Cluster A and Cluster B were compared. Statistical differences between the two subtypes were compared by Wilcoxon test (***P < 0.001). **(B, C)** The violin plots demonstrate the difference between TIDE score **(B)** and TIS score **(C)** in the Cluster A and Cluster B groups. Wilcoxon test was used to compare the differences between groups (***P < 0.001). **(D)** Submap analysis shows that Cluster A subtype could be more sensitive to anti-PD-1 treatment (Bonferroni corrected P-value = 0.00).

Since STS samples in clusters A and B show significant differences in TIME, we postulated differential responses to immunotherapy. Therefore, we analyzed the treatment responses of both molecular subtypes to immunotherapy by estimating the gene signatures related to immune checkpoint genes. Cluster A samples showed higher activation levels compared to the Cluster B samples for the immune checkpoint gene set (**Figure 4A**). This suggested that STS patients in Cluster A group would benefit more from the treatment with immune checkpoint inhibitors compared to the STS patients in Cluster B. Then, we verified these results using the TIS and TIDE scores. TIS score was higher and TIDE score was lower for the STS patients in Cluster A compared to the patients in Cluster B (**Figures 4B**, **C**). Finally, we predicted the responses of the two molecular subtypes to PD-1 and CTLA4 immune checkpoint inhibitors by Submap analysis. The results demonstrated that patients in Cluster A were more sensitive to anti-PD-1 therapy compared to the patients in Cluster B (**Figure 4D**).

### Analysis of DEGs shows two geneClusters of STS patients

**Figure 5:**
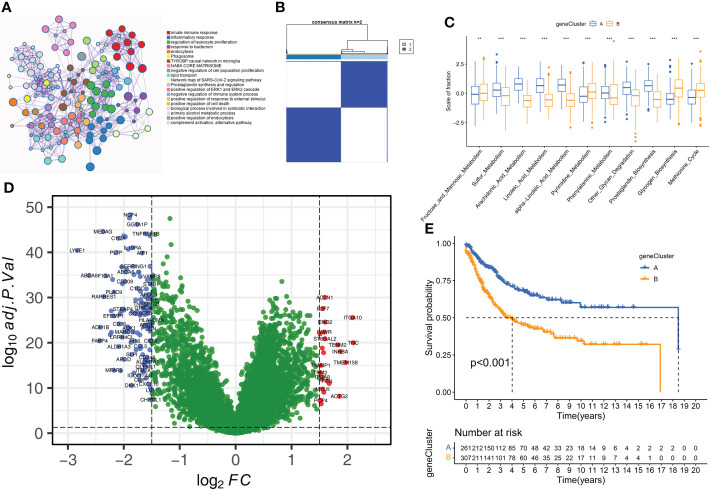
Construction of geneClusters. **(A)** Metascape enrichment network displayed the enriched terms for DEGs. Cluster annotations were shown in the color code. **(B)** The consensus clustering matrix for geneCluste (K=2). **(C)** The difference of 11 m6A related metabolic pathways between geneClusters (**P < 0.01; ***P < 0.001). **(D)** The volcano plot shows DEGs of between geneCluster-A and geneCluster-B. **(E)** Kaplan-Meier curves for overall survival of STS patients between geneCluster A and geneCluster B groups.

Next, we analyzed the DEGs between the two molecular subtypes by identifying the key genes and determine the potential biological effects. We identified 150 DEGs between the two molecular subtypes by analyzing gene expression profiles using the “limma” package (**Supplementary Table S4**). The result of the GO enrichment analysis was observed in the genes related to humoral immune response, complement activation, regulation of immune effect process, and some immune diseases (**Figure 5A**). Subsequently, unsupervised clustering analysis based on the expression patterns of the DEGs identified two molecular subtypes because K=2 was the optimal setting to distinguish the STS samples accurately (**Figures 5B**, **Supplementary Figure S3D**). The 568 STS samples were divided into geneCluster A (n=261) and geneCluster B (n=307). The 11 prognostic m6A-related metabolic pathways were significantly different between the two geneClusters (**Figure 5C**). Furthermore, 150 DEGs were between geneCluster A and geneCluster B were shown in **Figure 5D**. Kaplan-Meier survival curve analysis showed that the OS rates were higher for patients in geneCluster A compared to the patients in geneCluster B (**Figure 5E**). Thus, geneCluster A corresponds to the Cluster A phenotype, and geneCluster B corresponds to the Cluster B phenotype.

### The m6A-related metabolic scoring system was established for estimating prognosis of STS patients based on 12 hub genes

**Figure 6:**
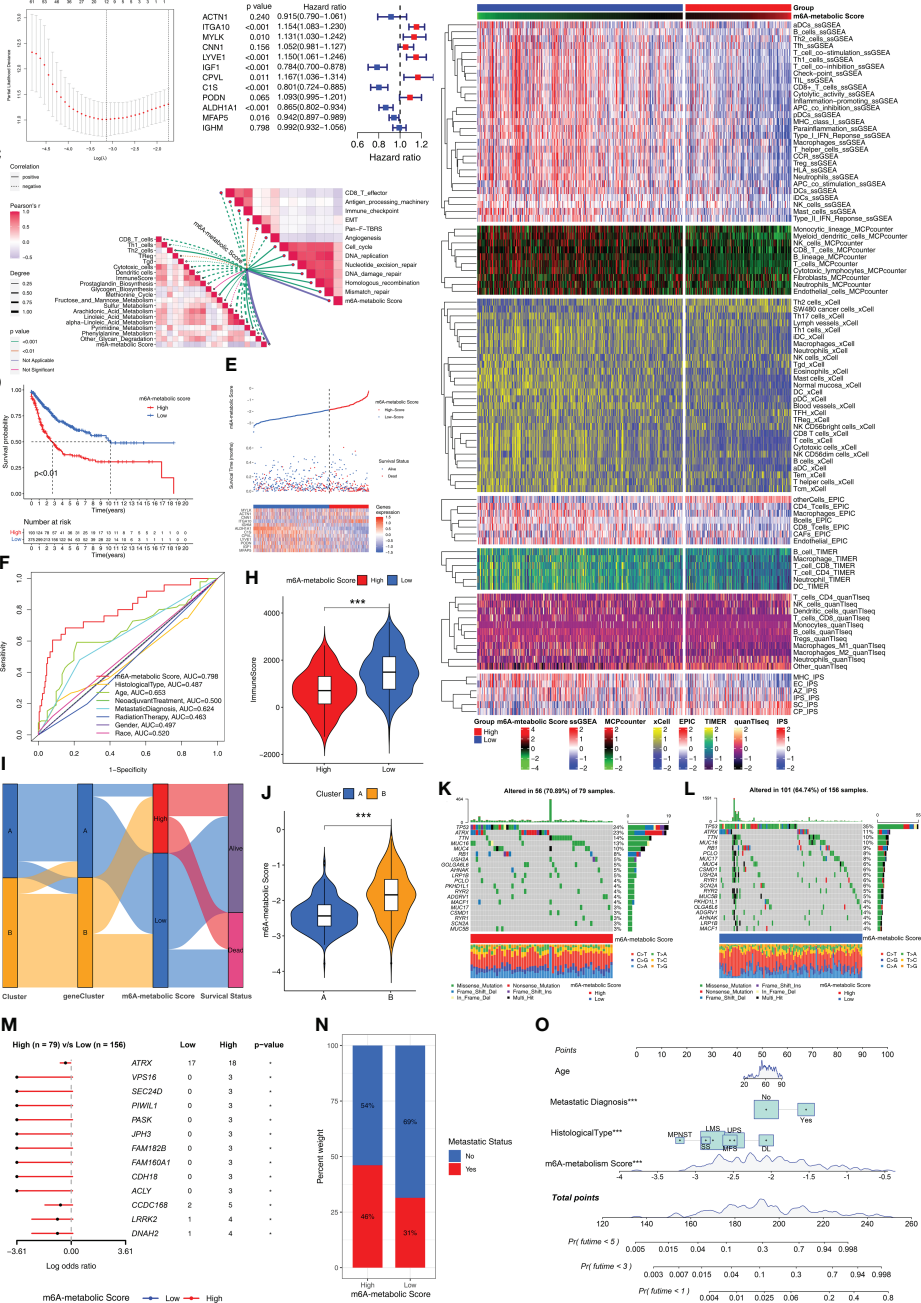
Biological characteristics and mutations between high and low m6A-metabolic Scores. **(A)** LASSO Cox regression model construction. λ selection by 10-fold cross-validation. The partial likelihood deviance with changing of log (λ) was plotted. **(B)** Forest plot shows 12 hub genes using multivariate cox regression analysis. **(C)** Correlations between m6A-metabolic Score and immune related biological processes, metabolic pathways, tumor-associated pathways. **(D)** Kaplan-Meier curves of overall survival in high and low m6A-metabolic Score groups for the TCGA-SARC and GSE21050 cohorts. **(E)** Comparison of differences in m6A metabolic Score, overall survival, and gene expression of 12 selected hub genes between high and low m6A-metabolic Score groups. **(F)** The ROC curve was used to calculate the area under the curve (AUC) of m6A-metabolic Score (AUC=0.798). **(G)** The heatmap demonstrates immune cell infiltration of high and low m6A-metabolic Score groups by ssGSEA, MCPcounter, xCell, EPIC, TIMER, quanTlseq and IPS algorithms. **(H)** The violin plot shows differences in immuneScore between the high and low m6A-metabolic Score groups. Statistical differences between the high and low m6A-metabolic Score groups were compared by the Wilcoxon test (***, P < 0.001). **(I)** Alluvial diagram displays changes in Clusters, geneClusters, m6A-metabolic Score and survival outcomes. **(J)** The violin plot reveals the differences of m6A-metabolic Scores between Cluster A and Cluster B The Wilcoxon test was used to compare the statistical difference between two groups. **(K, L)** The OncoPrint plots were drawn to reflect the landscape of mutations in high and low m6A-metabolic Score groups. **(M)** The forest plot revealed differences in 13 mutated genes between high and low m6A-metabolic Score groups (*, P < 0.05). **(N)** The proportion of tumor metastasis status in the high and low m6A-metabolic Score groups. Metastasis, red; No metastasis, blue. **(O)** A prognostic nomogram predicting 1-, 3-, and 5-year overall survival of STS.

We established a scoring system based on m6A-related metabolism (m6A-metabolic Score) to assess the prognosis of all STS patients (**Supplementary Table S5**). Firstly, we performed univariate Cox regression analysis for the 150 DEGs to identify the prognostic genes. Then, we identified 104 DEGs that were significantly associated with the OS rates of STS patients (**Supplementary Table S6**). Finally, a machine learning model, LASSO-penalized Cox analysis was used to identify 12 hub genes (*ACTN1, ITGA10, MYLK, CNN1, LYVE1, IGF1, CPVL, C1S, PODN, ALDH1A1, MFAP5*, and *IGHM*) for estimating the m6A-metabolic Score of each STS patients (**Figure 6A**). Among these hub genes, *ITGA10, MYLK, LYVE1, IGF1, CPVL, C1S, ALDH1A1* and *MFAP5* were identified as independent prognostic factors for the STS patients using multivariate Cox regression analysis (**Figure 6B**).

### Low m6A-metabolic scores group show better prognosis than high m6A-metabolic scores

Next, we analyzed the correlation between the m6A-metabolic score and TIME-related biological processes. The m6A-metabolic score showed negative correlation with CD8^+^ T effectors, antigen processing machinery, immune checkpoints, and other immune-related biological processes (**Figure 6C**). Then, based on the optimal cutoff m6A-metabolic score of -1.86, the STS samples were divided into two groups with high (n=193) and low (n=375) m6A-metabolic Scores. Kaplan-Meier survival curve analysis showed that the OS rates of STS patients with low m6A-metabolic Scores were significantly higher than those with high m6A-metabolic Scores (**Figure 6D**). The survival analysis was performed separately with the TCGA-SARC and GSE21050 cohorts to validate the accuracy of the results and the same trend was observed (**Supplementary Figures S3E, F**). These results demonstrated that the m6A-metabolic Score could be used to predict the survival of STS patients.

The distribution of m6A-metabolic Scores, survival status, survival time, and expression of the 12 hub genes are shown in **Figure 6E**. We then verified the accuracy in predicting prognosis of STS patients with m6A-metabolic Scores and obtained an AUC value of 0.798 in ROC curve (**Figure 6F**), thereby demonstrating the accuracy of the m6A-metabolic Score in predicting the survival outcomes of STS patients compared to the other clinicopathological indicators (Age, Histological type, Gender and Race, etc.). We then evaluated the differences in the immune cell proportions in the TME of the two groups using ssGSEA, MCPcounter, xCell, EPIC, TIMER, quanTIseq, and IPS algorithms. The results showed significantly higher infiltration of the anti-tumor immune cells such as CD8+ T cells, NK cells and dendritic cells in the low m6A-metabolic Score group compared to the high-m6A-metabolic Score group; the infiltration of pro-tumorigenic immune cells such as Th2 cells was higher in the high m6A-metabolic Score group compared to the low m6A-metabolic Score group (**Figure 6G**). ESTIMATE analysis also showed higher infiltration of the immune cells in the low m6A-metabolic Score group compared to the high m6A-metabolic Score group (**Figure 6H**).

The Cluster A and geneCluster A groups were associated with low m6A-metabolic Scores and better prognosis compared to the Cluster B and geneCluster B groups (**Figures 6I**, **J** and **Supplementary Figure S3G**). Furthermore, the mutation profiles and landscape were visualized using OncoPrint plot between the high- and low-m6A-metabolic Score groups. The high m6A-metabolic Scores group (**Figure 6K**) exhibited higher overall gene mutation rates than low m6A-metabolic Scores group (**Figure 6L**). As shown in **Figure 6M**, top 13 significant differences were observed in the high- and low-m6A-metabolic Score groups. The number of mutations were significantly higher in the high m6A-metabolic Score group compared to the low m6A-metabolic Score group. Furthermore, STS patients in the high m6A-metabolic Score group showed higher proportion of metastases compared to those in the low m6A-metabolic Score group (**Figure 6N**). Patients with complete clinical data were used to construct the prognostic nomogram to predict the 1-, 3-, and 5-year survival rates (**Figure 6O**).

We calculated m6A-metabolic Scores for different immune microenvironment tissue subtypes and found that the Inflammatory subtype had the lowest m6A-metabolic Score (**Supplementary Figure S4A**). We also found that patients in high ImmuneScore group had lower m6A-metabolic Scores than low ImmuneScore group (**Supplementary Figure S4B**). Next, according to the median survival time of STS patients, patients greater than the median were classified as longer survival time group, and those less than the median were classified as shorter survival time group. We found that the longer survival time group had lower m6A-metabolic Scores than the shorter survival time group (**Supplementary Figure S4C**). For patient survival status information, we also found that patients from alive group had lower m6A-metabolic Scores than dead group (**Supplementary Figure S4D**). Besides, STS patients with metastases had higher m6A-metabolic Scores than primary STS patients (**Supplementary Figure S4E**).

### STS patients with low m6A-metabolic scores are more sensitive to immunotherapy and chemotherapeutic drug sensitivity

**Figure 7:**
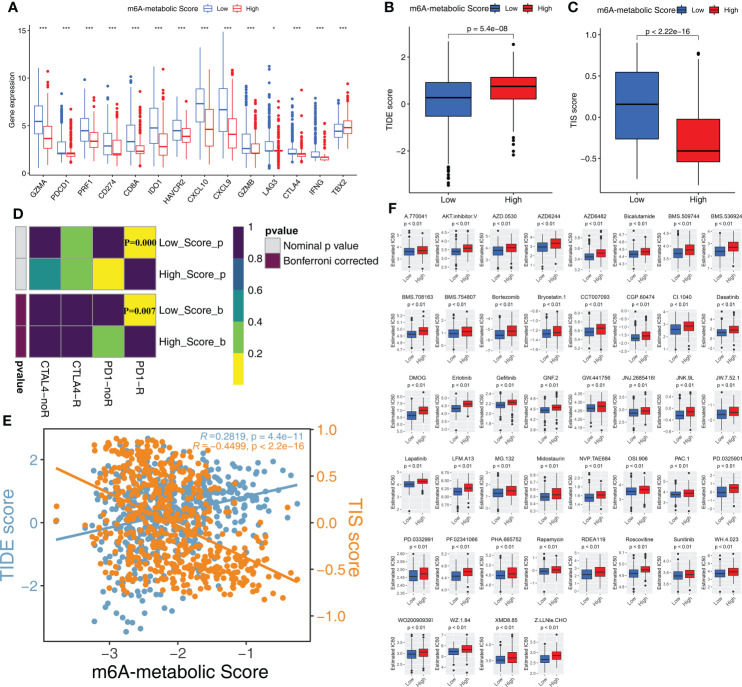
Immunotherapy response and drug sensitivity in high and low m6A-metabolic Score. **(A)** Differences in the gene expression of 14 immune checkpoint related genes in high and low m6A-metabolic Score groups. The thick line exhibited the median value. Statistical differences were compared by the Wilcoxon test (*P < 0.05; ***P < 0.001). **(B, C)** TIDE score **(B)** and TIS score **(C)** differences in the high and low m6A-metabolic Score groups. The upper and lower ends of the boxes indicated an interquartile range of values, the lines in the boxes represented median value. The statistical difference of two groups was compared through the Wilcoxon test. **(D)** Submap analysis shows that low m6A-metabolic Score groups could be more sensitive to anti-PD-1/PD-L1 treatment (Bonferroni corrected P-value = 0.007). **(E)** Correlation analysis (Spearman correlation) of m6A-metabolic Score with TIDEscore and TISscore. TIDE score, blue; TIS score, yellow. **(F)** The difference of chemotherapy response for 44 drugs between high and low m6A-metabolic Score groups. The statistical difference of two groups was compared through the Wilcoxon test.

The expression levels of 14 immune checkpoint-associated genes including PDCD1 and CD274 were significantly higher in the low m6A-metabolic Score group compared to the high m6A-metabolic Score group (**Figure 7A**). Furthermore, TIDE scores were lower and TIS scores were higher for the low m6A-metabolic Score group (**Figures 7B**, **C**). SubMap analysis showed that STS patients in the low m6A-metabolic Score group were more responsive to treatment with the PD-1 inhibitors (**Figure 7D**). The m6A-metabolic score also showed positive correlation with the TIDE score and negative correlation with the TIS score (**Figure 7E**). These suggested that STS patients with lower m6A-metabolic Scores were more sensitive to immunotherapy.

Chemotherapy is a standard treatment for the STS patients. Therefore, we analyzed the response of the two groups to chemotherapy. IC50 values indicate the potency of chemotherapeutic drugs to induce tumor cell apoptosis. STS samples in the low m6A-metabolic Score group were associated with lower IC50 values for 44 chemotherapeutic drugs compared with high m6A-metabolic Score group (**Figure 7F**). This suggested that chemotherapeutic response was significantly higher in the STS patients from the low m6A-metabolic Score group compared to the high m6A-metabolic Score group.

## Discussion

RNA methylation has been proven to play a significant role in several key physiological processes. Therefore, alterations in m6A RNA methylation are implicated in human pathology and play a key role in cancers, immune system diseases, neurological diseases, and others (31). Previous studies have demonstrated that m6A mRNA methylation regulates tumor immunity, metabolism, and stemness (10). However, the role of m6A mRNA methylation in the regulation of the TME through modulation of metabolic pathways and its effects on the prognosis and immunotherapeutic responses of STS patients is not widely reported. Therefore, in this study, we analyzed the multi-omics data from STS patients to determine the relationship between m6A mRNA methylation and the status of the metabolic pathways in the STS tissues and their impact on tumor immunity, progression, and prognosis. The multi-omics data analysis revealed two distinct and stable subtypes in the STS samples based on m6A-related metabolism. Furthermore, we established a scoring system based on the m6A-related metabolism. We demonstrated that the m6A-metabolic Scores can accurately predict the prognosis of STS patients and their response to immunotherapy.

We identified 11 m6A-related metabolic pathways that were associated with the prognosis of STS, including prostaglandin biosynthesis, glycogen biosynthesis, methionine cycle, fructose and mannose metabolism, sulfur metabolism, arachidonic acid metabolism, linoleic acid metabolism, alpha-linoleic acid metabolism, pyrimidine metabolism, phenylalanine metabolism, other glycan degradation. Among them, pyrimidine metabolic pathway promotes tumor progression by increasing cancer cell proliferation (32, 33). The pyrimidine metabolic pathway is also associated with drug resistance in various cancer patients (34). Linoleic acid metabolic pathway promotes proliferation and migration of breast cancer cells (35). Glycogen biosynthesis is also associated with breast cancer, bladder cancer, and others (36). The hypoxic environment in the tumor tissue promotes proliferation of breast cancer cells by activating the glycogen metabolism pathway. Our results suggested that hypoxia probably altered metabolism in the TME and contributed to the heterogeneity in the STS tissues (37). Besides, the activation of glycogen metabolism has been reported to promote aerobic glycolysis or the “Warburg” effect in the cancer cells, glycogen metabolism is a common metabolic pathway in cancer cells and is a significant marker of malignant tumors (38). Other metabolic pathways have been previously reported to be involved in tumorigenesis and progression. In this study, these important metabolic pathways were observed to be closely related to STS, which will provide new insights and evidence for future metabolic therapies for STS (39–41).

We further analyzed significance of the 11 m6A-related metabolic pathways in altering the tumor microenvironment and their relationship with prognosis in STS patients. Clustering analysis revealed two subtypes among the STS patients (Cluster A and Cluster B) based on the status of the m6A-related metabolic pathways. These two subtypes showed significant differences in OS and biological characteristics. The OS rates of the STS patients in Cluster A were significantly higher than those in Cluster B. These two molecular subtypes of STS also showed distinct profiles of metabolic pathways. STS tissues in Cluster A showed higher expression of genes involved in linoleic acid metabolism, alpha linoleic acid metabolism, prostaglandin biosynthesis, and arachidonic acid metabolism. These metabolic pathways improved the prognosis of STS patients in Cluster A.

The TIME plays a crucial role in the progression and prognosis of multiple cancers. Activation of innate and adaptive immunity increases the survival rates of cancer patients and their sensitivity to immunotherapy. Therefore, we compared the characteristics of the TIME between clusters A and B. We observed enrichment of tumor-suppressing immune cells such as CD8^+^ T effector cells in the Cluster A subtype, whereas, pro-tumorigenic pathways such as EMT were enriched in the Cluster B subtype. Furthermore, we used GSVA to analyze the differences in the enriched biological processes between Cluster A and Cluster B. The results showed significant enrichment of DNA replication in Cluster B, thereby suggesting increased proliferation of cancer cells. In Cluster A, we observed significant enrichment of the B cell receptor signaling pathway and natural killer (NK) cell mediated cytotoxicity, thereby suggesting enhanced activation of the immune cells. STS patients in Cluster B showed increased rate of metastasis and higher degree of malignancy compared to those in Cluster A. The higher survival rates of STS patients in Cluster A correlated with higher immune scores and increased infiltration to tumor-killing immune cells. In contrast, tumor-promoting immune cells were enriched in the Cluster B patients with STS and were associated with poor prognosis.

Previous studies have reported glucose and lipid metabolism plays a significant role in cancer stem cells originating from various cancers (42). Cancer stem cells rely highly on glucose and lipid metabolism for keeping their stemness features and satisfying their energy requirements, ultimately leading to tumor invasion and metastasis (43). Our results demonstrated that the tumor stemness levels was higher for STS tissues from Cluster B and was associated with increased metastasis. In addition, we explored the effects of alterations in tumor metabolism on EMT, which is often related with acquisition of stemness characteristics. Cluster B showed higher EMT scores, which supported increased incidence of tumor metastasis (44). Furthermore, compared with the Cluster A group, m6A regulators that were associated with metabolism in the STS tissues were up-regulated in Cluster B. Moreover, Cluster B group showed increased glycogen biosynthesis, methionine cycle, and pyrimidine metabolism. These data demonstrated that the m6A regulators modulated immune cell infiltration as well as tumor cell proliferation and progression by altering tumor cell metabolism. Furthermore, Cluster B was associated with higher rates of somatic mutations and higher degree of malignancy.

We also investigated the differences in immunotherapeutic responses between the STS patients in Cluster A and Cluster B. STS patients in Cluster A were more sensitive to immunotherapy. This suggested potential clinical application of the classification system based on m6A-related metabolic pathways. Therefore, we developed a scoring system (m6A-metabolic Score) for the STS patients based on m6A-related metabolism. This scoring system was based on the expression of 12 hub genes including eight hub genes (*ITGA10, MYLK, LYVE1, IGF1, CPVL, C1S, ALDH1A1* and *MFAP5*) that were identified as independent prognostic biomarkers of STS. The m6A-metabolic Score was significantly lower for STS patients in Cluster A compared to those in Cluster B. Our data suggested that the m6A-metabolic Score was a reliable tool for comprehensive assessment of the two molecular subtypes based on m6A-related metabolism and could be used to determine the status of tumor immune infiltration and patient survival outcomes. We also demonstrated that the m6A-metabolic Score was a robust tool for determining the efficacy of immune checkpoint inhibitors in individual STS patients.

In conclusion, our study performed a comprehensive analysis of the multi-omics data from STS patients and classified them into two molecular subtypes based on m6A-related metabolism. We also established a m6A-related metabolism scoring system and demonstrated its accuracy in predicting the prognosis of the STS patients and predicting their response to immunotherapy. Therefore, the m6A-metabolic Score shows great promise in clinical application for accurately classifying STS patients at a molecular level and may be used as a guide for precision therapy of individual STS patients. However, our study also has several limitations. Firstly, we integrated two large STS cohorts for our analysis. This may have masked the heterogeneity in different cohorts. We corrected potential batch effects to overcome this issue. Secondly, the m6A-metabolic Score cutoff value requires further validation in larger cohorts of STS patients.

## Data availability statement

All data used in this work can be acquired from the Gene-Expression Omnibus (GEO; https://www.ncbi.nlm.nih.gov/geo/) and the GDC portal (https://portal.gdc.cancer.gov/). The accession number(s) can be found in the article/**supplementary material**.

## Author contributions

Z-DH, CH, and R-XW conceived and designed this study. Z-DH, R-XW and Y-CF carried out the analysis procedure. Z-DH, S-YL and Y-JM analyzed the results. Z-DH, Y-CF, S-YL and YZ contributed analysis tools. Z-DH, Y-CF, CH and R-XW participated in the manuscript writing and revising. Z-DH and R-XW mainly participated in the manuscript revising. All authors contributed to the article and approved the submitted version.

## Conflict of interest

The authors declare that the research was conducted in the absence of any commercial or financial relationships that could be construed as a potential conflict of interest.

## Publisher’s note

All claims expressed in this article are solely those of the authors and do not necessarily represent those of their affiliated organizations, or those of the publisher, the editors and the reviewers. Any product that may be evaluated in this article, or claim that may be made by its manufacturer, is not guaranteed or endorsed by the publisher.

## Supplementary material

Supplementary Table 1 Clinical information for STS samples in the TCGA-SARC and GSE21050 cohorts.

Supplementary Table 2 The gene sets used in this study including 12 oncogenic pathways.

Supplementary Table 3 35 metabolic pathways correlated with m6A regulators (the absolute value of the correlation coefficient was greater than 0.4).

Supplementary Figure 1 Sample distributions from TCGA and GEO before batch effect correction. The “ComBat” algorithm was applied to reduce the likelihood of batch effects from non-biological technical biases between TCGA-SARC and GSE21050 cohorts.

Supplementary Figure 2
**(A)** Relative change in area under consensus cumulative distribution functions (CDF) curve (k = 2-9) using consensus clustering based on 11 m6A related metabolic pathways for total patients of TCGA-SARC and GSE21050 cohorts. **(B)** The consensus matrix heatmap corresponding to k=2-9 obtained by consensus clustering.

Supplementary Figure 3
**(A)** Relative change in area under consensus CDF curve (k = 2-9) using consensus clustering based on m6A related metabolic pathways for TCGA-SARC (left) and GSE21050 (right) cohorts, respectively. **(B)** The heatmap demonstrates differences in 11 m6A-related metabolic pathways scores between Cluster A and Cluster B. **(C)** The heatmap demonstrates immune cell infiltration of two molecular subtypes from unsupervised clustering in the TCGA-SARC and GSE21050 cohorts by ssGSEA, MCPcounter, xCell, EPIC, TIMER, quanTlseq and IPS algorithms. **(D)** Relative change in area under consensus CDF curve (k = 2-9) using consensus clustering based on DEGs for total population of TCGA-SARC and GSE21050 cohorts. **(E, F)** Survival analyses for the two Clusters in **(E)** TCGA-SARC and **(F)** GSE21050 cohorts, respectively. **(G)** The violin plot reveals the differences of m6A-metabolic Scores between geneCluster A and geneCluster B.

Supplementary Figure 4
**(A)** The boxplot reveals the differences of m6A-metabolic Scores between between STS patients with different immune microenvironment tissue subtype. **(B-E)** These boxplots reveal the differences of m6A-metabolic Scores between between STS patients with different ImmuneScore level **(B)**, survival time **(C)**, survival status **(D)**, metastatic status **(E)**.

## References

[1] Dammerer D VANB A Schneeweiss V Schwabegger A. Follow-up strategies for primary extremity soft-tissue sarcoma in adults: A systematic review of the published literature. In Vivo (Athen Greece) (2020) 34(6):3057–68. doi: 10.21873/invivo.12140 PMC7811670 33144410

[2] Ayodele O Razak ARA. Immunotherapy in soft-tissue sarcoma. Curr Oncol (Toronto Ont) (2020) 27(Suppl 1):17–23. doi: 10.3747/co.27.5407 PMC7050043 32174754

[3] Comandone A Petrelli F Boglione A Barni S. Salvage therapy in advanced adult soft tissue sarcoma: A systematic review and meta-analysis of randomized trials. Oncol (2017) 22(12):1518–27. doi: 10.1634/theoncologist.2016-0474 PMC5728024 28835514

[4] Perrier A Didelot A Laurent-Puig P Blons H Garinet S. Epigenetic mechanisms of resistance to immune checkpoint inhibitors. Biomolecules (2020) 10(7):1061. doi: 10.3390/biom10071061 PMC7407667 32708698

[5] Judge SJ Darrow MA Thorpe SW Gingrich AA O'Donnell EF Bellini AR. Analysis of tumor-infiltrating NK and T cells highlights IL-15 stimulation and TIGIT blockade as a combination immunotherapy strategy for soft tissue sarcomas. J Immunother Cancer (2020) 8(2):e001355. doi: 10.1136/jitc-2020-001355 33158916 PMC7651745

[6] Martín-Broto J Moura DS Van Tine BA. Facts and hopes in immunotherapy of soft-tissue sarcomas. Clin Cancer Res (2020) 26(22):5801–8. doi: 10.1158/1078-0432.ccr-19-3335 PMC7669707 32601077

[7] Roulleaux Dugage M Nassif EF Italiano A Bahleda R. Improving immunotherapy efficacy in soft-tissue sarcomas: A biomarker driven and histotype tailored review. Front Immunol (2021) 12:775761. doi: 10.3389/fimmu.2021.775761 34925348 PMC8678134

[8] He L Li H Wu A Peng Y Shu G Yin G. Functions of N6-methyladenosine and its role in cancer. Mol Cancer (2019) 18(1):176. doi: 10.1186/s12943-019-1109-9 31801551 PMC6892141

[9] Han X Wang L Han Q. Advances in the role of M(6)A RNA modification in cancer metabolic reprogramming. Cell Biosci (2020) 10:117. doi: 10.1186/s13578-020-00479-z 33062255 PMC7552565

[10] Huang ZD Lin LL Liu ZZ Hu C Gu HY Wei RX. M6a modification patterns with distinct immunity, metabolism, and stemness characteristics in soft tissue sarcoma. Front Immunol (2021) 12:765723. doi: 10.3389/fimmu.2021.765723 35003079 PMC8739240

[11] Xia L Oyang L Lin J Tan S Han Y Wu N. The cancer metabolic reprogramming and immune response. Mol Cancer (2021) 20(1):28. doi: 10.1186/s12943-021-01316-8 33546704 PMC7863491

[12] Thorsson V Gibbs DL Brown SD Wolf D Bortone DS Ou Yang TH. The immune landscape of cancer. Immunity (2018) 48(4):812–30.e14. doi: 10.1016/j.immuni.2018.03.023 29628290 PMC5982584

[13] Mayakonda A Lin DC Assenov Y Plass C Koeffler HP. Maftools: Efficient and comprehensive analysis of somatic variants in cancer. Genome Res (2018) 28(11):1747–56. doi: 10.1101/gr.239244.118 PMC6211645 30341162

[14] Mermel CH Schumacher SE Hill B Meyerson ML Beroukhim R Getz G. GISTIC2.0 facilitates sensitive and confident localization of the targets of focal somatic copy-number alteration in human cancers. Genome Biol (2011) 12(4):R41. doi: 10.1186/gb-2011-12-4-r41 21527027 PMC3218867

[15] Rosario SR Long MD Affronti HC Rowsam AM Eng KH Smiraglia DJ. Pan-cancer analysis of transcriptional metabolic dysregulation using the cancer genome atlas. Nat Commun (2018) 9(1):5330. doi: 10.1038/s41467-018-07232-8 30552315 PMC6294258

[16] Wang T Kong S Tao M Ju S. The potential role of RNA N6-methyladenosine in cancer progression. Mol Cancer (2020) 19(1):88. doi: 10.1186/s12943-020-01204-7 32398132 PMC7216508

[17] Wilkerson MD Hayes DN. ConsensusClusterPlus: A class discovery tool with confidence assessments and item tracking. Bioinf (Oxf Engl) (2010) 26(12):1572–3. doi: 10.1093/bioinformatics/btq170 PMC2881355 20427518

[18] Sturm G Finotello F List M. Immunedeconv: An r package for unified access to computational methods for estimating immune cell fractions from bulk RNA-sequencing data. Methods Mol Biol (Clifton NJ) (2020) 2120:223–32. doi: 10.1007/978-1-0716-0327-7_16 32124323

[19] Hänzelmann S Castelo R Guinney J. GSVA: Gene set variation analysis for microarray and RNA-seq data. BMC Bioinf (2013) 14:7. doi: 10.1186/1471-2105-14-7 PMC3618321 23323831

[20] Yoshihara K Shahmoradgoli M Martínez E Vegesna R Kim H Torres-Garcia W. Inferring tumour purity and stromal and immune cell admixture from expression data. Nat Commun (2013) 4:2612. doi: 10.1038/ncomms3612 24113773 PMC3826632

[21] Wu T Hu E Xu S Chen M Guo P Dai Z. Clusterprofiler 4.0: A universal enrichment tool for interpreting omics data. Innovation (New York NY) (2021) 2(3):100141. doi: 10.1016/j.xinn.2021.100141 PMC8454663 34557778

[22] Malta TM Sokolov A Gentles AJ Burzykowski T Poisson L Weinstein JN. Machine learning identifies stemness features associated with oncogenic dedifferentiation. Cell (2018) 173(2):338–54.e15. doi: 10.1016/j.cell.2018.03.034 29625051 PMC5902191

[23] Ritchie ME Phipson B Wu D Hu Y Law CW Shi W. Limma powers differential expression analyses for RNA-sequencing and microarray studies. Nucleic Acids Res (2015) 43(7):e47. doi: 10.1093/nar/gkv007 25605792 PMC4402510

[24] Friedman J Hastie T Tibshirani R. Regularization paths for generalized linear models *via* coordinate descent. J Stat Softw (2010) 33(1):1–22. doi: 10.18637/jss.v033.i01 20808728 PMC2929880

[25] Jiang P Gu S Pan D Fu J Sahu A Hu X. Signatures of T cell dysfunction and exclusion predict cancer immunotherapy response. Nat Med (2018) 24(10):1550–8. doi: 10.1038/s41591-018-0136-1 PMC6487502 30127393

[26] Danaher P Warren S Lu R Samayoa J Sullivan A Pekker I. Pan-cancer adaptive immune resistance as defined by the tumor inflammation signature (TIS): Results from the cancer genome atlas (TCGA). J Immunother Cancer (2018) 6(1):63. doi: 10.1186/s40425-018-0367-1 29929551 PMC6013904

[27] Hoshida Y Brunet JP Tamayo P Golub TR Mesirov JP. Subclass mapping: Identifying common subtypes in independent disease data sets. PLoS One (2007) 2(11):e1195. doi: 10.1371/journal.pone.0001195 18030330 PMC2065909

[28] Roh W Chen PL Reuben A Spencer CN Prieto PA Miller JP. Integrated molecular analysis of tumor biopsies on sequential CTLA-4 and PD-1 blockade reveals markers of response and resistance. Sci Trans Med (2017) 9(379):eaah3560. doi: 10.1126/scitranslmed.aah3560 PMC5819607 28251903

[29] Gu Z Eils R Schlesner M. Complex heatmaps reveal patterns and correlations in multidimensional genomic data. Bioinf (Oxf Engl) (2016) 32(18):2847–9. doi: 10.1093/bioinformatics/btw313 27207943

[30] Geeleher P Cox N Huang RS. Prrophetic: An r package for prediction of clinical chemotherapeutic response from tumor gene expression levels. PLoS One (2014) 9(9):e107468. doi: 10.1371/journal.pone.0107468 25229481 PMC4167990

[31] Jiang X Liu B Nie Z Duan L Xiong Q Jin Z. The role of M6a modification in the biological functions and diseases. Signal Transduct Target Ther (2021) 6(1):74. doi: 10.1038/s41392-020-00450-x 33611339 PMC7897327

[32] Edwards L Gupta R Filipp FV. Hypermutation of DPYD deregulates pyrimidine metabolism and promotes malignant progression. Mol Cancer Res (2016) 14(2):196–206. doi: 10.1158/1541-7786.mcr-15-0403 26609109 PMC5024535

[33] Wang X Yang K Wu Q Kim LJY Morton AR Gimple RC. Targeting pyrimidine synthesis accentuates molecular therapy response in glioblastoma stem cells. Sci Trans Med (2019) 11(504):eaau4972. doi: 10.1126/scitranslmed.aau4972 PMC7568232 31391321

[34] Gu X Tohme R Tomlinson B Sakre N Hasipek M Durkin L. Decitabine- and 5-azacytidine resistance emerges from adaptive responses of the pyrimidine metabolism network. Leukemia (2021) 35(4):1023–36. doi: 10.1038/s41375-020-1003-x PMC7867667 32770088

[35] Gonzalez-Reyes C Marcial-Medina C Cervantes-Anaya N Cortes-Reynosa P Salazar EP. Migration and invasion induced by linoleic acid are mediated through fascin in MDA-MB-231 breast cancer cells. Mol Cell Biochem (2018) 443(1-2):1–10. doi: 10.1007/s11010-017-3205-8 29052029

[36] Massari F Ciccarese C Santoni M Iacovelli R Mazzucchelli R Piva F. Metabolic phenotype of bladder cancer. Cancer Treat Rev (2016) 45:46–57. doi: 10.1016/j.ctrv.2016.03.005 26975021

[37] Tang K Zhu L Chen J Wang D Zeng L Chen C. Hypoxia promotes breast cancer cell growth by activating a glycogen metabolic program. Cancer Res (2021) 81(19):4949–63. doi: 10.1158/0008-5472.can-21-0753 34348966

[38] Shulman RG Rothman DL. The glycogen shunt maintains glycolytic homeostasis and the warburg effect in cancer. Trends Cancer (2017) 3(11):761–7. doi: 10.1016/j.trecan.2017.09.007 29120752

[39] Yarla NS Bishayee A Sethi G Reddanna P Kalle AM Dhananjaya BL. Targeting arachidonic acid pathway by natural products for cancer prevention and therapy. Semin Cancer Biol (2016), 40–41:48–81. doi: 10.1016/j.semcancer.2016.02.001 26853158

[40] Locasale JW. Serine, glycine and one-carbon units: Cancer metabolism in full circle. Nat Rev Cancer (2013) 13(8):572–83. doi: 10.1038/nrc3557 PMC3806315 23822983

[41] Ward NP DeNicola GM. Sulfur metabolism and its contribution to malignancy. Int Rev Cell Mol Biol (2019) 347:39–103. doi: 10.1016/bs.ircmb.2019.05.001 31451216

[42] Visweswaran M Arfuso F Warrier S Dharmarajan A. Aberrant lipid metabolism as an emerging therapeutic strategy to target cancer stem cells. Stem Cells (Dayton Ohio) (2020) 38(1):6–14. doi: 10.1002/stem.3101 31648395

[43] Yi M Li J Chen S Cai J Ban Y Peng Q. Emerging role of lipid metabolism alterations in cancer stem cells. J Exp Clin Cancer Res (2018) 37(1):118. doi: 10.1186/s13046-018-0784-5 29907133 PMC6003041

[44] Kim BN Ahn DH Kang N Yeo CD Kim YK Lee KY. TGF-β induced EMT and stemness characteristics are associated with epigenetic regulation in lung cancer. Sci Rep (2020) 10(1):10597. doi: 10.1038/s41598-020-67325-7 32606331 PMC7326979

