# Supplementary figures and images for: m6A-related metabolism molecular classification with distinct prognosis and immunotherapy response in soft tissue sarcoma
Source: Front Immunol. 2022 Jul 28;13:895465. doi: 10.3389/fimmu.2022.895465 (PMC9374037; doi:10.3389/fimmu.2022.895465)

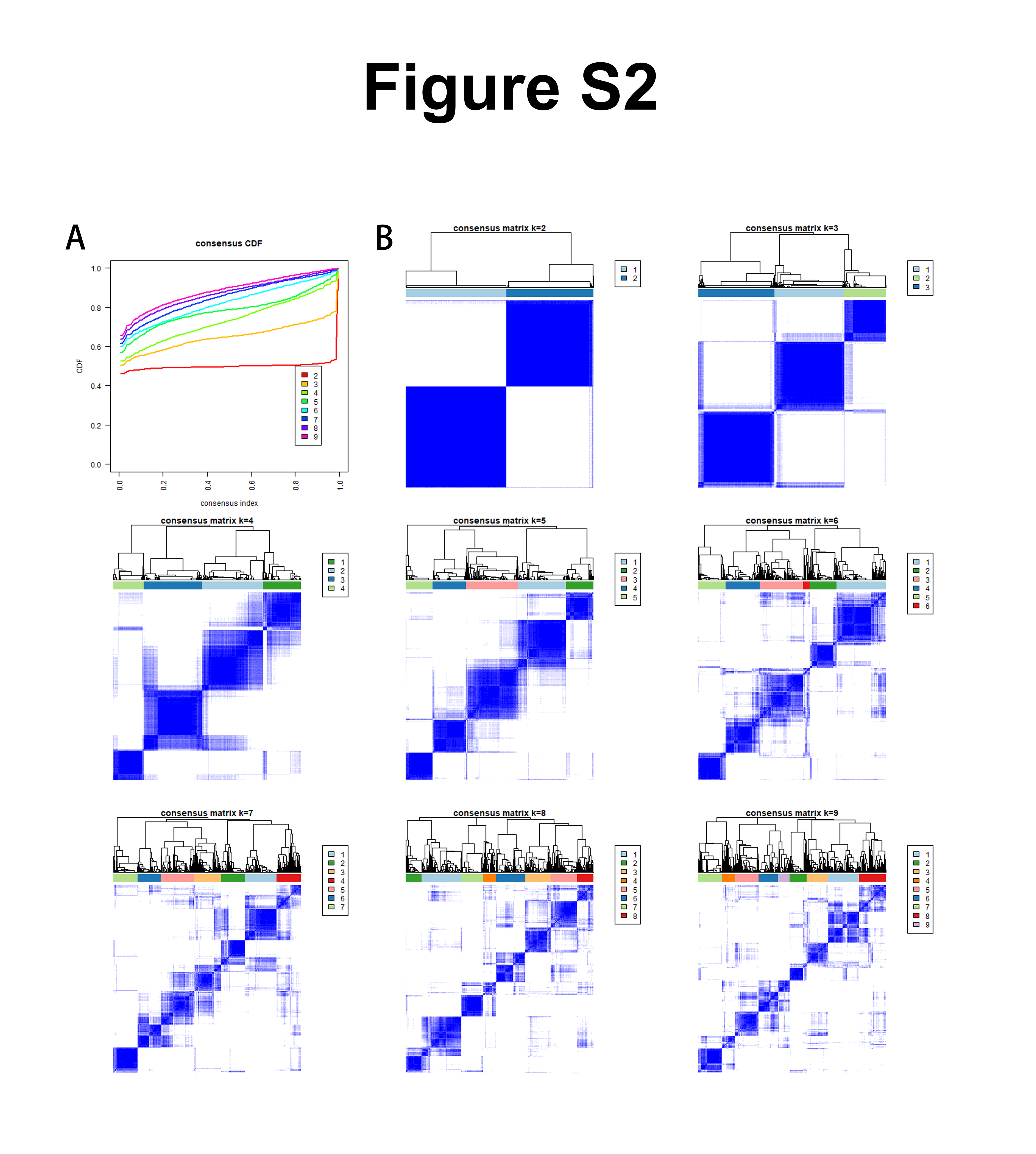

Supplement: Supplementary Figure 2 — (A) Relative change in area under consensus cumulative distribution functions (CDF) curve (k = 2-9) using consensus clustering based on 11 m6A related metabolic pathways for total patients of TCGA-SARC and GSE21050 cohorts. (B) The consensus matrix heatmap corresponding to k=2-9 obtained by consensus clustering. [file Image_2.tif]

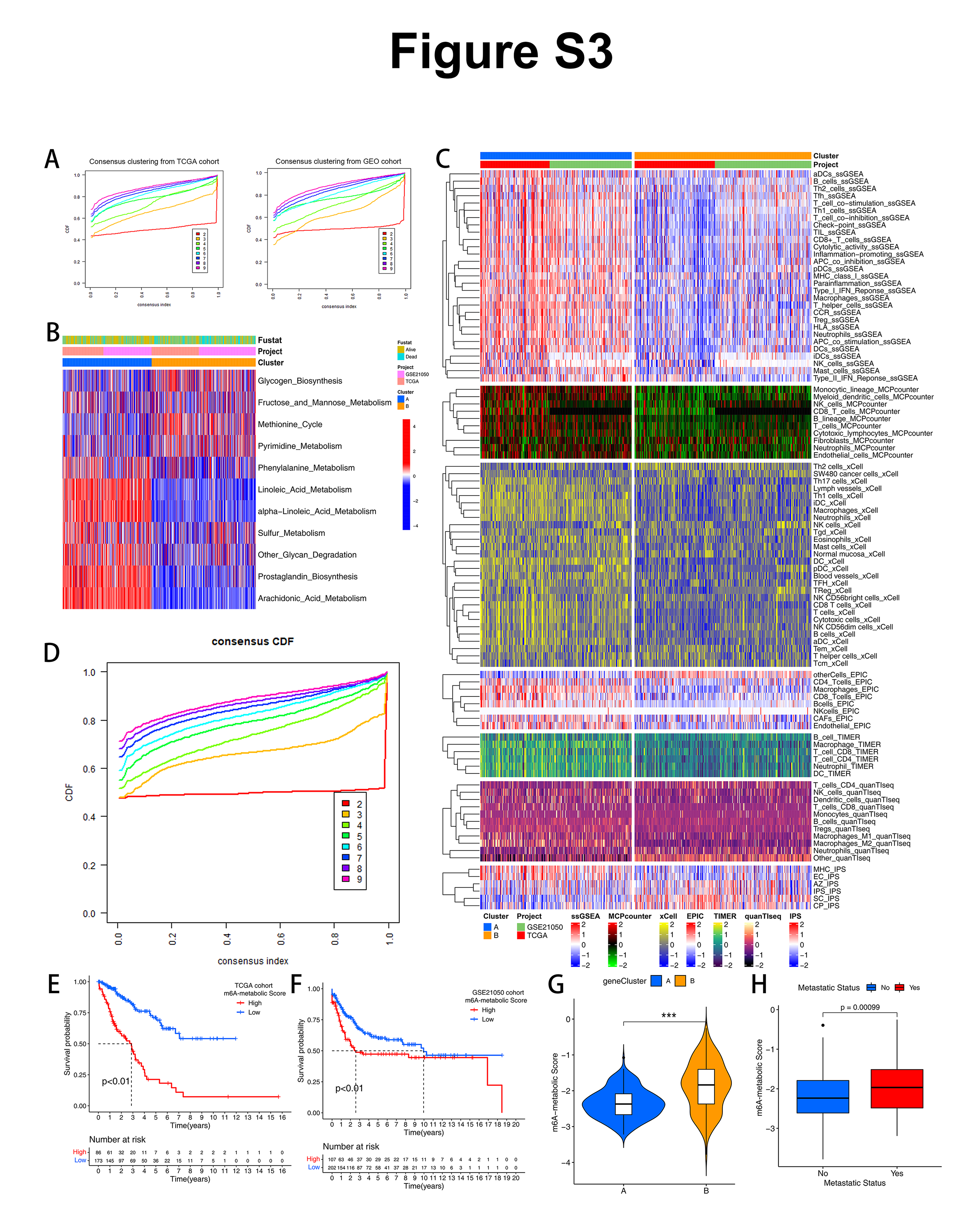

Supplement: Supplementary Figure 3 — (A) Relative change in area under consensus CDF curve (k = 2-9) using consensus clustering based on m6A related metabolic pathways for TCGA-SARC (left) and GSE21050 (right) cohorts, respectively. (B) The heatmap demonstrates differences in 11 m6A-related metabolic pathways scores between Cluster A and Cluster B. (C) The heatmap demonstrates immune cell infiltration of two molecular subtypes from unsupervised clustering in the TCGA-SARC and GSE21050 cohorts by ssGSEA, MCPcounter, xCell, EPIC, TIMER, quanTlseq and IPS algorithms. (D) Relative change in area under consensus CDF curve (k = 2-9) using consensus clustering based on DEGs for total population of TCGA-SARC and GSE21050 cohorts. (E, F) Survival analyses for the two Clusters in (E) TCGA-SARC and (F) GSE21050 cohorts, respectively. (G) The violin plot reveals the differences of m6A-metabolic Scores between geneCluster A and geneCluster B. [file Image_3.tif]

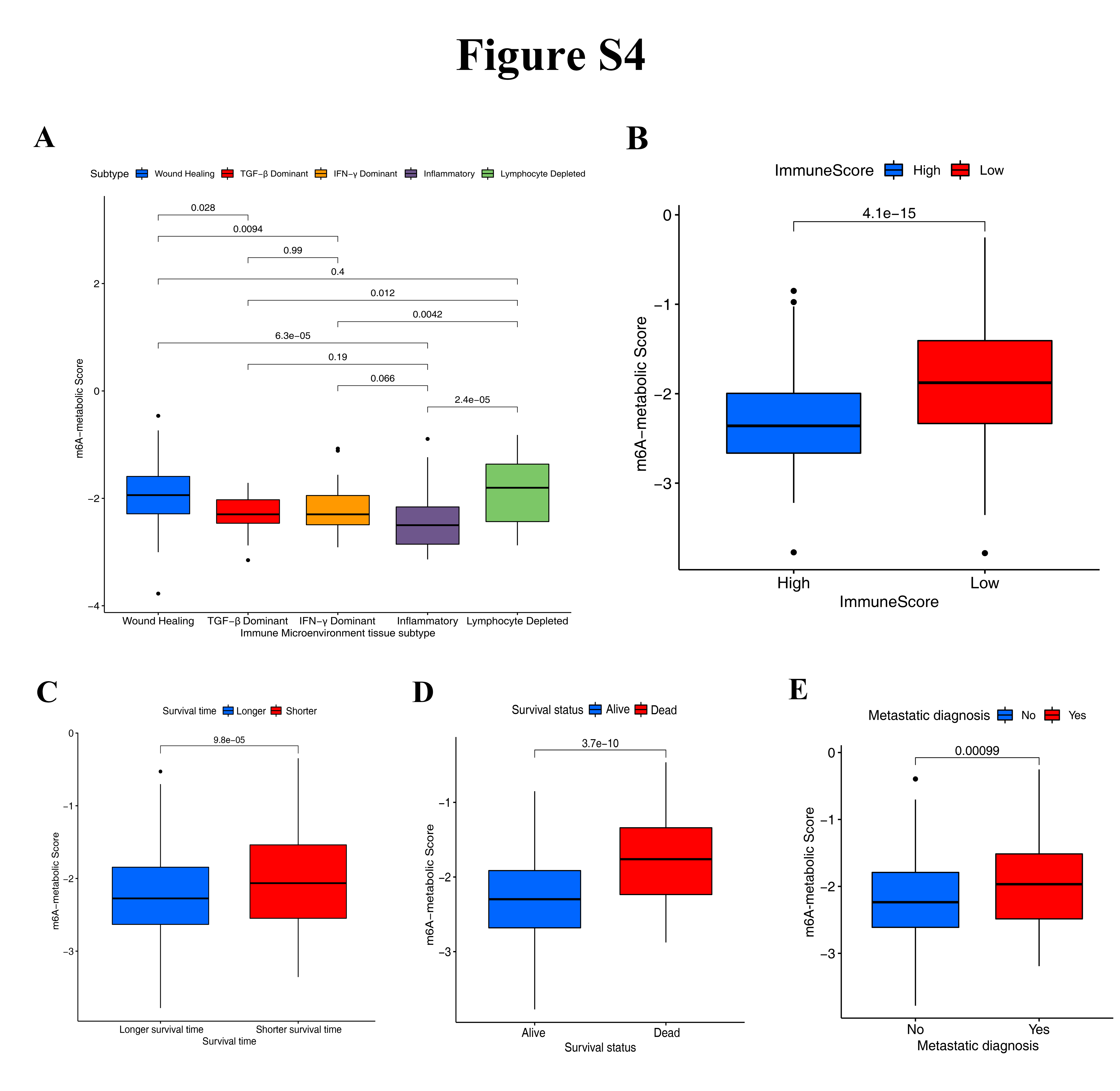

Supplement: Supplementary Figure 4 — (A) The boxplot reveals the differences of m6A-metabolic Scores between between STS patients with different immune microenvironment tissue subtype. (B-E) These boxplots reveal the differences of m6A-metabolic Scores between between STS patients with different ImmuneScore level (B), survival time (C), survival status (D), metastatic status (E). [file Image_4.tif]
